# Orthodontic findings in adults with Trisomy 21

**DOI:** 10.1007/s00784-024-05846-5

**Published:** 2024-07-30

**Authors:** Susanne Wriedt, Fabienne Service, Irene Schmidtmann, Christina Erbe

**Affiliations:** 1 Department of Orthodontics, University Medical Center of the Johannes Gutenberg-University, Augustusplatz 2, 55131 Mainz, Germany; 2 Institute of Medical Biostatistics, Epidemiology and Informatics, Johannes Gutenberg-University Medical Center, Mainz, Germany

**Keywords:** Trisomy 21, Down syndrome, Orthodontic findings, Adult patients, Epidemiology

## Abstract

**Objectives:**

Objective of this study was to describe orthodontic findings in adults with Down’s syndrome, a matter insufficiently regarded in literature.

**Materials and methods:**

A group of 104 adults (33.8 ± 15 years) with trisomy 21 had an orthodontic check-up in their accustomed environment. Anamnestic and dental findings completed the examination and descriptive analysis was performed using SPSS23. Relative frequencies with 95% confidence intervals were compared to the average population (SHIP-study, 2003; DMS IV, 2006).

**Results:**

Among the participants 46.2% (36.3–56.2%) (SHIP 36.7%) had already undergone orthodontic treatment. In 87.5% (79.6–93%) of the patients, less than 25.6 properly functioning permanent teeth (DMS IV’s mean) were found. Gingival bleeding and recessions, as well as periodontal disease, were increasingly found in older affected persons. Patients with Down’s syndrome showed less crowding, e.g., maxillary incisors 28% (19.3–39%) versus 41.9% (SHIP). Frontal open bite (35.2% (25.3–46.1%) versus 3.6% (SHIP)) and frontal crossbite (40.9% (30.5–51.9%) versus 4.2% (SHIP)) were more often observed. No considerable differences in frequencies of orthodontic findings were detected in the comparison of the subgroups “18–28 years” versus “>28 years”, “with” versus “without orthodontic treatment”, “male” versus “female”, “with” versus “without periodontal problems”, or “with” versus “without orofacial disturbances”.

**Conclusions:**

Within the bounds of this study, we gathered orthodontic findings in adults with trisomy 21 for the first time. In comparison to the average population, the subject group showed a greater number of complex orthodontic findings.

**Clinical relevance:**

These persisting dental and orofacial problems must be considered when treating patients with Down’s syndrome.

## Introduction

Life expectancy for people with Down’s syndrome (DS) has increased dramatically in recent decades thanks to advances in medical intervention and therapy [1]. This is reflected in an exponential increase since 1900, as the life expectancy of people with trisomy 21 approaches the expectations of the general population [2]. Consequently, more age-related diseases are occurring today, requiring a rethinking of special health care needs.

**Fig. 1:**
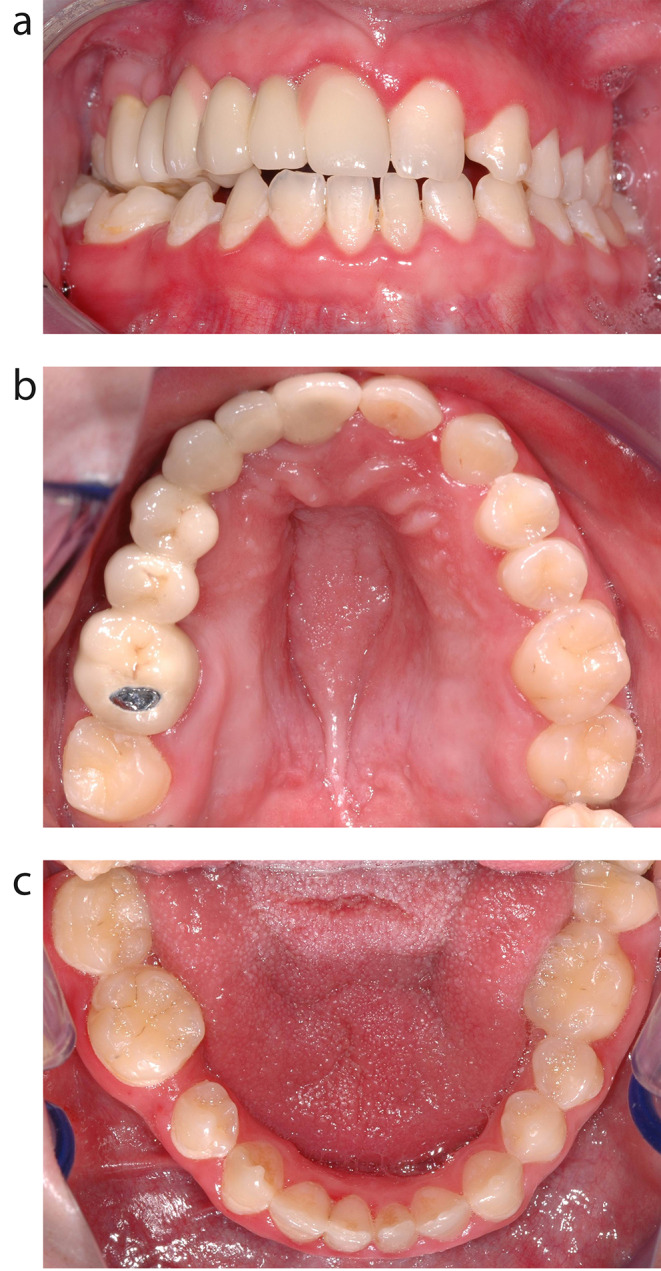
a-c: Intraoral photos of a 21-year-old man with DS. Despite orthodontic treatment during childhood and following prosthetic rehabilitation one can detect a smaller maxilla and a broader mandibula leading to a lateral crossbite- and an open-bite-tendency. There is an aplasia of the teeth 15, 13, 12, 22, 45. Gingivitis with tendency toward bleeding and periodontal disease can be seen in the upper front

In addition to general health conditions, oral health problems are common in people with DS. These primarily include periodontal disease, malocclusions, mouth breathing, delayed tooth eruption, hypodontia, bruxism, and dental aplasia [3]. Malocclusions, depending on their degree of severity, lead to difficulties in speech, mastication, and swallowing [4]. Intraoral photos of a 21-year-old man with DS are shown in Fig. 1a-c. Despite orthodontic treatment during childhood and following prosthetic rehabilitation one can detect a smaller maxilla and a broader mandibula leading to a lateral crossbite- and an open-bite-tendency. There is an aplasia of the teeth 15, 13, 12, 22, 45. Gingivitis with tendency toward bleeding and periodontal disease can be seen in the upper front.

The dental and skeletal abnormalities, as well the orofacial dysfunction in children with trisomy 21 have already been investigated worldwide [4–6]. The studies uniformly prove that malocclusions of the teeth occur frequently in this patient population, usually requiring treatment via an interdisciplinary therapy concept.

In the 1980s and 1990s, the education of dentists shifted towards the treatment of disabled patients. The paediatrician Castillo Morales had already developed his therapy for neuromotor development as well as the orofacial regulation therapy (ORT) in the 1970s [4]. Over the years, the observation and early therapy of orofacial function in disabled children also received increasing attention in Germany. The ORT includes special exercises of physical therapy to rise the muscular tonus of the orofacial muscles as well as stimulation of the orofacial muscles by little knobbles on an orthodontic plate during time without exercises.

However, clinical studies collecting data concerning the frequency of dental and orthodontic findings in adults with DS are rare [3, 7]. Some study data are available from other cultural settings [8–11], but the number of patients studied is often small. Usually, children and adults are studied together. In some cases, data were collected as part of a study of generally mentally and/or physically disabled people, and therefore results regarding only the group with DS cannot be differentiated [12, 13].

The aim of this study is to describe orthodontic findings in patients with DS. These findings have been missing in the literature so far, thus this study provides a targeted assessment of the orthodontic treatment need in adults with trisomy 21.

## Materials and methods

This study was approved by the Ethics Committee of the Medical Chamber of Rhineland Palatinate (9618). We interviewed and examined adult patients with DS in their usual environment. Inclusion criteria were trisomy 21, the patient’s/caregiver’s consent to the study, and participants had to be 18 years and older. Patients of any gender and ethnicity were eligible to participate. Exclusion criteria were lack of compliance, lack of informed consent, or severe general illness.

To recruit subjects for this study, 30 public and private institutions for people with disabilities in the Rhine-Main area were contacted between May 2015 and November 2017. In addition, further subjects were recruited and examined during their treatment at the University Medical Center Mainz or through personal private contacts.

Written informed consent was obtained from the patient’s caregivers. The examination of the subjects occurred at the place of residence (parents, home for disabled persons) in 40.4% of the cases, in 26% at the workplace, in 18.3% during leisure activities, and in 15.3% during a visit to the dentist.

Two examiners performed the clinical examination. SW has more than 30 years of experience as an orthodontist and is trained in scientific studies. She trained and calibrated FS. FS examined 100 patients; SW looked at four patients. During the examination, we used oral mirrors and wooden spatula. A headlamp illuminated the orofacial region, as no other person assisted. No periodontal probes or other instrument were used, as some of the patients were very frightened being examined.

The dentition findings (number of permanent and deciduous teeth) as well as the orthodontic (crossbites, overbite, overjet, crowding or spacing), periodontal (bleeding, swelling), and functional findings (open mouth posture, swallowing pattern, tongue position) were collected from the subjects. In addition, the caregivers were interviewed regarding dental and orthodontic history. No additional radiographs were taken.

Deciduous and permanent teeth and their position in the dentition were determined. The absence of “posterior crossbite” was defined as the contact of the buccal cuspid of the mandibular tooth to the occlusal plane of the maxillary molar. The incidence of posterior crossbite was seen, if there was a contact of the buccal cuspid of the maxillary posterior teeth to the occlusal sulcus of the mandibular teeth. Posterior crossbite can occur uni- or bilateral. The vertical distance of the maxillary and mandibular incisors is called “overbite”, the sagittal distance “overjet”. Both show a conventional value of 2–3 mm. “Open bite” was diagnosed, if there was no contact in the vertical dimension between the maxillary and mandibular teeth in the frontal or posterior region. “Frontal crowding” was defined as broken contacts between at least three incisors followed by lack of space for correct aligning of the incisors. More than 2 mm space between the incisors was listed as “Frontal spacing”. “Gingival recession” was defined as the visibility of the cemento-enamel limit without inflammation. Bleeding, redness, and/or swelling of the gingival margin were counted as sign of periodontal disease. “Open mouth posture” was noted if the patient did not close their lips during rest-time, e.g. while waiting. “Swallowing pattern” was noted as “somatic” or “visceral”, depending on the position of the tongue either in the palatal vault or against the incisors while swallowing a sip of water. The caregivers were interviewed regarding therapy with the palatal plate for simulation of the orofacial muscles and/or usual orthodontic treatment.

The analysis of this study is for an exploratory data collection. Categorical variables are presented descriptively with absolute and relative frequencies so that the prevalence of individual findings can be estimated. The prevalence of individual findings is described by reporting the relative frequency, and exact 95% confidence intervals are provided for this purpose. With a patient number of *n* = 104, 95% confidence intervals can be determined, their limits not exceeding more than 10% (percentage points) away from the prevalence estimated in this sample and with a coverage probability of at least 90%. Subgroups of our sample are compared by performing the Chi²-test. The prevalence of the collected findings of the present study is compared with the data in the literature from the Study of Health in Pomerania (SHIP 0) and a study with DS children [5, 14].

Microsoft^®^ Excel^®^ for Windows 2007 (version 12.0.6) and IBM^®^ SPSS^®^ Statistics software (version 23 V5) were used for the statistical analysis and charting.

## Results

Of the regarded 104 adults with DS, 55 were male and 49 were female. The age of the participants ranged from 18 to 78 years with a mean age of 33.8 ± 15 years. Of the subjects 87.5% (CI 95% = 79.6–93.2%) showed less than 25.6 permanent teeth, which marks the mean of existent permanent teeth in the population of DMS IV [15]. At least one deciduous tooth was still existent among 30 patients; especially deciduous second molars and lateral incisors were to be found.

Among the patients, 44.2% reported having a check-up with their general dentist twice or more per year. And while 39.4% of participants showed signs of periodontal disease, 46.2% of patients reported receiving or having received orthodontic treatment. Among them, 32.4% underwent treatment with the Castillo-Morales’ palatal plate as infants, 22% had been treated with removable appliances, and 24% with fixed orthodontic appliances. The frequencies of the orthodontic findings are shown in the right column of Table 1 (Adults with DS). Concerning orofacial dysfunction, 43 (41.3%) of the DS patients had an open mouth posture. A visceral swallowing pattern was exhibited in 60 (57.7%) and 64 (61.5%) of the patients had their tongues constantly positioned behind their teeth.

**Table 1:** Orthodontic findings: comparison of frequencies in mean population [14], children with DS [5]and adults with DS (present study). Avg. = average; CI = confidence interval

frequency [%]	mean population	children with DS	adults with DS [avg (CI)]
frontal crowding maxilla	41.9	N/A	28 (19.3–39)
frontal crowding mandibula	62.9	N/A	27.3 (18.3–37.8)
frontal spacing maxilla	15.1	N/A	37.5 (27.4–48.5)
frontal spacing mandibula	9.2	N/A	35.2 (25.3–46.1)
overbite < 0 mm	3.6	16.7	35.2 (25.3–46.1)
overbite = 0 mm	5.9	N/A	33 (23.3–43.8)
increased overbite (> 3 mm)	23.8	N/A	14.8 (8.1–23.9)
frontal crossbite	4.2	66.7	40.9 (30.5–51.9)
overjet = 0 mm	1.1	N/A	23.9 (15.4–34.1)
overjet > 4 mm	36.8	N/A	4.5 (1.3–11.2)
crossbite right side	14.8	N/A	51.7 (40.8–62.4)
crossbite left side	14.9	N/A	55.7 (44.7–66.3)
open bite right side	1.1	N/A	31.8 (22.3–42.6)
open bite left side	0.9	N/A	33 (23.3–43.8)
orthodontic treatment	26.7	N/A	46.2 (36.3–56.2)

We classified the patients into two groups concerning different parameters: “with” versus “without orthodontic treatment”, “male” versus “female”, “with” versus “without periodontal problems”, or “with” versus “without orofacial disturbances”. The Chi²-test indicated no statistically significant differences between the results of the different divisions. We also undertook a classification according to age: 49 patients ranged from 18–28 years and 55 patients were older than 28 years. The different orthodontic findings of the two age groups are shown in table 2.

**Table 2:** Comparison of orthodontic and dental findings in patients with DS: aged18-28 years versus > 28 years; avg. = average; CI = confidence interval

frequency [%; avg (CI)]	18–28 years (47.1%)	> 28 years (52.9%)
frontal crowding maxilla	32.7 (19.9–47.6)	23.1 (11.1–39.3)
frontal crowding mandibula	28.6 (16.6–43.3)	25.6 (13.0-42.1)
frontal spacing maxilla	40.8 (27.0-55.8)	33.3 (19.1–50.2)
frontal spacing mandibula	38.8 (25.2–53.8)	30.8 (17.0-47.6)
overbite < 0 mm	44.9 (30.7–59.8)	23.1 (11.1–39.3)
overbite = 0 mm	20.4 (10.2–34.3)	48.7 (32.4–65.2)
increased overbite	16.3 (7.3–29.7)	12.8 (4.3–27.4)
frontal crossbite	40.8 (27.0-55.8)	41 (25.6–57.9)
overjet = 0 mm	24.5 (13.3–38.9)	23.1 (11.1–39.3)
overjet > 4 mm	8.2 (2.3–19.6)	0 (0–0)
crossbite right side	44.9 (30.7–59.8)	60 (43.3–75.1)
crossbite left side	51 (36.3–65.6)	61.5 (44.6–76.6)
open bite right side	34.7 (21.7–49.6)	28.2 (15-44.9)
open bite left side	34.7 (21.7–49.6)	30.8 (17-47.6)
orthodontic treatment	85.7 (72.8–94.1)	18.9 (4.1–22.2)
palatal plate for stimulation	49 (34.4–63.7)	18.2 (9.1–30.9)
gingival recession	2 (0.1–10.9)	29.1 (17.6–42.9)
bleeding/periodontal disease	32.7 (19.5–47.5)	41.8 (28.7–55.9)

## Discussion

Most international studies on dental problems connected to the DS recruit data from the patients’ medical files. Thus, it cannot be avoided that a certain pre-selection of patients is made, as they are often referred to such institutions for the treatment of e.g., severe malocclusions, oligodontia, cleft lip and palate or stimulation plate therapy. In the present study, unlike other similar studies, most participants were not recruited directly through a medical school. Instead, contact was made predominantly through residential and work communities. Therefore, it can be assumed that the results shown here are more likely to represent the actual population with DS. Nevertheless, a slight selection bias should be considered in the present study, because only study participants whose caregivers consented to the study were included. Consequently, subjects who developed poor compliance due to frequent visits to the doctor at a young age were omitted. Furthermore, all those people with DS who are dependent on intensive nursing care and do not live in a public institution could not be represented.

Efforts were made to ensure a non-distressing examination for the subject. Hence, we relied solely on a headlamp and an oral mirror to avoid frightening the patients. We abstained from taking x-rays, as we consider studies in which x-rays are taken for the examination without a justifiable indication are ethically unacceptable.

Since the present study group is a patient population from Germany, the values of the German population surveys SHIP, and DMS IV are used for comparison. The SHIP study, among others, examined 1777 adults aged 20–49 years regarding their dental and jaw status [14]. Furthermore, we compared our group of adults with the values of a group of 30 children with DS (aged 8–14 years) [5].

It is noticeable that young adults with DS exhibit several missing teeth. Of the patients, 87.5% (79.6–93.2%) had less than 25.6 functional teeth (mean value of DMS IV) [15]. This poor state can be attributed, in addition to the more frequent noncompliance, to increased aplasia of teeth [16–18], and to increased tooth loss due to periodontal disease. The periodontal condition of adolescents and adults with DS was assessed in detail by Franz in 2002 [19]. In the present study, especially the older patients demonstrated increased gingival bleeding and periodontal diseases (41.8% (28.7–55.9%)). This results in an intensified treatment demand for preventive and therapeutic action. Dental professionals should strive for close monitoring and regular dental cleaning of this patient group [10].

Of the adults examined, 46.2% (36.3–56.2%) had a similar frequency of orthodontic treatment compared to the normal population (SHIP: 36.7%). However, the patients with DS displayed many more severe dental and skeletal differences than the average population. Patients with DS showed fewer crowding problems (e.g., upper anterior 28% (19.3–39%) vs. 41.9% (SHIP). Frontal open bites (35.2% (25.3–46.1%) / (SHIP 3.6%)) and frontal crossbite (40.9% (30.5–51.9%) / (SHIP 4.2%)) occurred more frequently.

In the vertical dimension, the prevalence of a frontal open bite is higher in children with DS [5]. The results of the present study show that despite orthodontic therapy, the frontal open bite often cannot be closed or recurs. This is due to the hypotonic tongue muscles and the often persisting visceral swallowing pattern. Concerning the orofacial dysfunctions, 43 (41.3%) of the adult patients with DS had an open mouth posture and 60 (57.7%) showed a visceral swallowing pattern. In 64 (61.5%) of the patients, the tongue was physiologically permanently behind the teeth, but overall orofacial dysfunctions seem to play a major role.

Frontal crossbite was slightly less frequent in adults with DS than in children. However, it is significantly more often compared to the general population. The reasons for this are the typical hypodontia and the persisting tongue dysfunction which can cause protrusion of the lower incisors. Likewise, lateral crossbites and lateral open bites occur more frequently in adults with DS. Overall, malocclusions are more common and more pronounced in DS patients [20]. In addition to malocclusions, orofacial dysfunctions can often be observed, which may amplify the dysgnathia. These include bruxism which can lead to an increased occurrence of abrasions and attritions of the teeth and can be observed more frequently in the present work. Orthodontic treatment seems to have a positive effect on the reduction of frontal and lateral crossbite as well as lateral open bite.

Improved care for disabled patients and the nationwide rollout of the Orofacial Regulation Therapy [4] took place about 28 years before the examination of our patients. Therefore, the circumstances of DS-treatment were changed, and we were now looking for an improvement of the dental outcome. But when comparing the groups 18–28 years/>28 years, there were no significant changes in the frequency of tooth and jaw malocclusions. This can be seen in the overlap of the corresponding confidence intervals. Though the younger group of adults with DS was statistically more likely to be treated with the stimulating palatal plate in early childhood and more likely to receive orthodontic treatment afterwards, the number of orthodontic malocclusions is still notably more pronounced in the younger group than in the average population, despite above-average orthodontic treatment frequency. The retention or recurrence of orofacial malfunctions seem to be partly responsible for a moderate long-term success of orthodontic treatment. A prospective emphasis on the continuation of logopaedic exercises should be considered. In any case, orthodontic treatment strategies not constricting the tongue-space (build-ups of hypoplastic teeth; avoiding extractions; temporary anchorage devices for mesialisation of teeth) are inevitable in this group of patients.

The consequences of untreated malocclusions lead to restrictions in chewing and speaking functions as well as oral hygiene. In order to include people with DS in society in the best possible way, greater influence should be exercised over these grievances. The results of this study, which show the higher prevalence of many malocclusions, should be proved by further multicenter studies and an even larger group of subjects, as well as long-term controls after documented treatment processes.

Even though malocclusions seem to have a high recurrence rate after orthodontic treatment in adults with DS, it is advisable to set the treatment goal for the individual optimum. This should primarily be aimed at supporting tooth preservation and oral health, as well as reducing dysfunctions. Tooth change in patients with DS is often delayed and compliance in infancy and adolescence is often limited. Therefore, a reconsideration by the health insurances to pay for orthodontic treatment of DS patients even beyond the age of 18 years is necessary. Improved orthodontic results are expected due to a more probable orofacial function. We expect an improvement in chewing function, articulation, and periodontal status of the teeth, which results in a higher quality of life and thus better inclusion in society. The treatment of malocclusions is therefore highly recommended. In this context, it is particularly important to ensure that the long-term result is stable, requiring close monitoring during the retention phase. Since speech therapy and ORT can further help prevent the high recurrence rate of malocclusions in adulthood, it would make sense to include this in a treatment concept for adults.

## Conclusion

Within the constraints of this study, we ascertained orthodontic findings in adults with trisomy 21 as a reference for the first time and further the inadequate database of orthodontic findings in adults with DS. The comparison of data with an average population study enables a more targeted approach to assess orthodontic treatment needs.

In summary, it can be stated that adults with DS have a significantly higher incidence of malocclusions of varying severity compared to the average population. Depending on compliance, orthodontic and speech therapy is also useful in adulthood to improve the patient’s quality of life. Sufficient space for the tongue seems significant and therefore extraction of teeth should be avoided and all teeth, even the deciduous, should be preserved if possible. Necessary closing of gaps can be done by building up hypoplastic teeth or mesialisation against temporary anchorage devices. To substantiate the results of this study, further multicenter studies with an even larger number of subjects should follow, as well as long-term controls after documented treatment courses.

## Data Availability

No datasets were generated or analysed during the current study.
